# A Simple and Non-destructive Method for Chlorophyll Quantification of *Chlamydomonas* Cultures Using Digital Image Analysis

**DOI:** 10.3389/fbioe.2020.00746

**Published:** 2020-07-21

**Authors:** Nicola J. Wood, Alison Baker, Rupert J. Quinnell, Miller Alonso Camargo-Valero

**Affiliations:** ^1^Centre for Doctoral Training in Bioenergy, School of Chemical and Process Engineering, University of Leeds, Leeds, United Kingdom; ^2^BioResource Systems Research Group, School of Civil Engineering, University of Leeds, Leeds, United Kingdom; ^3^Centre for Plant Sciences and School of Molecular and Cellular Biology, Faculty of Biological Sciences, University of Leeds, Leeds, United Kingdom; ^4^School of Biology, Faculty of Biological Sciences, University of Leeds, Leeds, United Kingdom; ^5^Departamento de Ingeniería Química, Universidad Nacional de Colombia, Manizales, Colombia

**Keywords:** chlorophyll, digital image analysis, RGB color model, microalgae, *C. reinhardtii*

## Abstract

Growing interest in the use of microalgae as a sustainable feedstock to support a green, circular, bio-economy has led to intensive research and development initiatives aimed at increasing algal biomass production covering a wide range of scales. At the heart of this lies a common need for rapid and accurate methods to measure algal biomass concentrations. Surrogate analytical techniques based on chlorophyll content use solvent extraction methods for chlorophyll quantification, but these methods are destructive, time consuming and require careful disposal of the resultant solvent waste. Alternative non-destructive methods based on chlorophyll fluorescence require expensive equipment and are less suitable for multiple sampling of small cultures which need to be maintained under axenic growth conditions. A simple, inexpensive and non-destructive method to estimate chlorophyll concentration of microalgal cultures *in situ* from digital photographs using the RGB color model is presented. Green pixel intensity and chlorophyll *a, b* and total chlorophyll concentration, measured by conventional means, follow a strong linear relationship (*R*^2^ = 0.985–0.988). In addition, the resulting standard curve was robust enough to accurately estimate chlorophyll concentration despite changes in sample volume, pH and low concentrations of bacterial contamination. In contrast, use of the same standard curve during nitrogen deprivation (causing the accumulation of neutral lipids) or in the presence of high quantities of bacterial contamination led to significant errors in chlorophyll estimation. The low requirement for equipment (i.e., a simple digital camera, available on smartphones) and widely available standard software for measuring pixel intensity make this method suitable for both laboratory and field-based work, particularly in situations where sample, qualified personnel and/or equipment is limited. By following the methods described here it should be possible to produce a standard curve for chlorophyll analysis in a wide range of testing conditions including different microalga cultures, culture vessel and photographic set up in any particular laboratory.

## Introduction

Photosynthetic microalgae have gained attention for their ability to efficiently convert solar energy into biomass as a potential source of biofuels and high-value chemicals (Chisti, 2007; Wijffels and Barbosa, 2010; Liu and Benning, 2013; Goncalves et al., 2016). Chlorophylls *a* and *b* are the primary photosynthetic pigments in microalgae and are responsible for the characteristic green color of chlorophyte cultures. The quantity of these pigments is therefore related to biomass production and can provide an indication of the growth of the culture (Wood et al., 2005). Additionally, unlike growth measurements such as optical density or dry biomass weight, chlorophyll content can be used as a measurement of culture density without interference from non-photosynthetic organisms such as bacterial contaminants. In industrial applications the production of neutral lipids (i.e., triacylglycerols, TAGs), which are favored for use as a biodiesel starting product, has been linked to breakdown and recycling of membrane lipids including the chloroplast membrane (Moellering and Benning, 2010; Siaut et al., 2011). The rapid and accurate determination of changes in chlorophyll content can therefore be used as an indicator of possible neutral lipid production in microalgal cultures.

Conventional methods for chlorophyll quantification involve destructive solvent extraction and subsequent spectroscopic chlorophyll analysis (Porra et al., 1989). These methods are time consuming, require removal of sample from the culture vessel and subsequent destruction of the sample and require careful disposal of the resultant solvent waste. The removal and destruction of sample can be problematic, in particular for time course studies, where culture volume can limit the number of measurable parameters.

Fluorescence is widely used as a non-destructive method of chlorophyll detection and quantification in both plants (Buschmann et al., 2000) and algae (Vincent, 1983), including in environmental samples (Wang et al., 2018), and commercial products are available (Netto et al., 2005). However, these require specialized expensive equipment and may not be suited to small volumes and to maintaining culture sterility. There is a need for a simple, inexpensive, non-destructive method that does not require multiple sampling of small volume cultures which need to be maintained axenically.

Recently, digital imaging techniques have emerged as a means by which to rapidly and non-destructively measure chlorophyll content. In particular, the almost universal presence of smartphones has enabled the use of smartphone cameras in laboratory digital analysis (Rignon et al., 2016). A handful of studies have made use of the RGB color model for chlorophyll determination in plant leaves such as maize (Friedman et al., 2016), potato plants (Gupta et al., 2013) and in marine microalgal cultures (e.g., *N. oculta*; Su et al., 2008) via digital imaging. The RGB color scale represents the intensity of red, green and blue components of a pixel within a digital computer image. Intensity ranges on a scale from 0 to 255 for each color, where white has RGB values (255,255,255) and black has values (0,0,0). However, many methods published to date require the use of advanced modeling software or complex mathematical processing (Su et al., 2008; Dey et al., 2016; Friedman et al., 2016).

In this study, the microalga *Chlamydomonas reinhardtii* has been used to develop a simple and rapid method to quantify chlorophyll concentration of algal cultures non-destructively *in situ* using the RGB scale to quantify pixel color intensity from digital photographs. The effect of bacterial contamination, sample volume, pH and neutral lipid production on the accuracy and reproducibility of the method is also evaluated. The method presented here is much simplified compared with other digital analysis methods for chlorophyll determination. Furthermore, the method is non-destructive and can be conducted without removal of sample from the culture vessel. This method requires minimal data manipulation and the use of only easily accessible software packages.

The method used to make the standard curve presented here is for monitoring chlorophyll concentrations in cultures of *C. reinhardtii* grown in 7 ml Bijou flasks. Digital images have been acquired under constant light conditions at a specific location using a smartphone digital camera. By creating a standard curve for the specific organism, culture conditions and photographic set-up available in any individual laboratory, the method presented here could be used to determine chlorophyll concentration for a range of algal species or consortia grown in a range of culture vessels.

## Materials and Methods

### Cultivation and Sample Preparation

*Chlamydomonas reinhardtii* strain CC-1690 (*wt, mt*+) was obtained from the Chlamydomonas Resource Center (2016) (University of Minnesota, USA; www.chlamycollection.org) and cultivated in sterile Tris-Acetate-Phosphate (TAP) media which contains 7.5 mM NH_4_Cl as the only available nitrogen source; the composition is detailed in Gorman and Levine (1965), cited in www.chlamycollection.org. Starter cultures were grown statically in 5 ml TAP media contained within 7 ml plastic Bijou flasks fitted with screw-top lids (Medline Scientific, Product Code 129202) at 20°C with a 16-h photoperiod (~50 μmol photons m^−2^ s^−1^).

All cultures were grown to stationary phase before being aliquoted, with a range of sample volumes, into fresh 7 ml Bijou flasks. Unless otherwise stated, all samples were made up to a total of 5 ml with TAP media, to create cultures with a range of absorbance values at 600 nm (A_600_) between 0.005 and 2.5, for chlorophyll analysis. The control dataset (sterile CC-1690, 5 ml, pH 7.0–8.5) samples were cultured as described and analyzed as described in sections Photographic Chlorophyll Analysis and Chlorophyll Analysis by Solvent Extraction, using 5 ml standard TAP media as a baseline.

In order to test the reproducibility of the analysis when environmental interference is present, a range of commonly encountered parameters were chosen for investigation; the altered culture and sample preparation methods are detailed in sections Testing the effect of sample volume, Testing the effect of high pH, Testing the effect of bacterial contamination, Testing the effect of induced lipid accumulation.

#### Testing the Effect of Sample Volume

“Low volume” samples (sterile CC-1690, 3.5 ml, pH 7.0–8.5) were cultured as described in section Cultivation and Sample Preparation but made up to a final volume of 3.5 ml with TAP media. Samples were analyzed as described in sections Photographic Chlorophyll Analysis and Chlorophyll Analysis by Solvent Extraction using 3.5 ml standard TAP media as a baseline.

#### Testing the Effect of High pH

“High pH” samples (sterile CC-1690, 5 ml, pH 9.5) were cultured as described in section Cultivation and Sample Preparation before being transferred in CAPS-Acetate-Phosphate media (TAP media containing 10 mM CAPS buffer in place of the Tris buffer, adjusted to pH 9.5 by addition of 1M KOH). Starter cultures were decanted into 1.5 ml Eppendorf tubes, pelleted (6,000 rpm, ~3,400 g, 5 min) in a microcentrifuge, washed twice in new media and resuspended in CAPS-Acetate-Phosphate media (pH = 9.5) to a range of biomass concentrations. Analysis was conducted as described in sections Photographic Chlorophyll Analysis and Chlorophyll Analysis by Solvent Extraction using 5 ml CAPS-Acetate-Phosphate media as a baseline.

Absorbance spectra at pH 7.0 and 9.5 were obtained by pelleting 2 × 500 μl samples from a stationary phase culture grown as described above. The supernatant was removed, and the samples resuspended in 1.5 ml TAP or CAPS-Acetate-Phosphate media, respectively. The samples were left at room temperature for 30 min before measuring the absorbance spectra in the visible range (400–750 nm). 1 ml of each sample was then taken for chlorophyll quantification as described in section Chlorophyll Analysis by Solvent Extraction.

#### Testing the Effect of Bacterial Contamination

Contaminated samples (CC-1690 + *E. coli*, 5 ml, pH 7.0–8.5) were created by the addition of two different amounts of stationary phase *Escherichia coli* (*E. coli*) culture (A_600_ = 3.64). The *E. coli* culture was decanted into 1.5 ml aliquots contained within 1.5 ml Eppendorf tubes and pelleted (13,000 rpm, ~16,000 g, 10 min) in a microcentrifuge, the supernatant removed and the cultures resuspended in TAP medium. Sixteen *C. reinhardtii* samples were prepared; each sample was prepared to one of eight optical densities (A_600_ 0.005–1.0) in duplicate. Each duplicate was spiked with 1.5 ml or 0.325 ml *E. coli* culture (approximate A_600_ = 1.0 and 0.25, respectively) to create a range of algal biomass concentrations containing two different *E. coli* contaminant concentrations. Analysis was conducted as described in sections Photographic Chlorophyll Analysis and Chlorophyll Analysis by Solvent Extraction using 5 ml TAP media as a baseline.

#### Testing the Effect of Induced Lipid Accumulation

Large quantities of neutral lipids are known to accumulate in microalgae under nitrogen starvation (Siaut et al., 2011; Valledor et al., 2014). “TAP-N” samples (sterile CC-1690, 5 ml, pH 7.0–8.5, TAP-N media) were created to test the effect of neutral lipid accumulation on photographic chlorophyll determination. Starter cultures were cultured as described in section Cultivation and Sample Preparation before being transferred into TAP-N media (TAP media omitting NH_4_Cl). Starter cultures were decanted into 1.5 ml Eppendorf tubes, pelleted (6,000 rpm ~3,400 g, 5 min) in a microcentrifuge, washed twice in new media and resuspended in TAP-N media to range of biomass concentrations. Analysis was conducted as described in sections Photographic Chlorophyll Analysis and Chlorophyll Analysis by Solvent Extraction using 5 ml TAP-N media as a baseline.

### Photographic Chlorophyll Analysis

Analysis was first conducted via the photographic digital analysis method demonstrated here, followed by comparison with a standard analytical method for chlorophyll quantification of microalgal cultures. A simplified step by step protocol for creating the standard curve is presented in the Supplementary Material.

#### Photographic Set-Up

**Figure 1:**
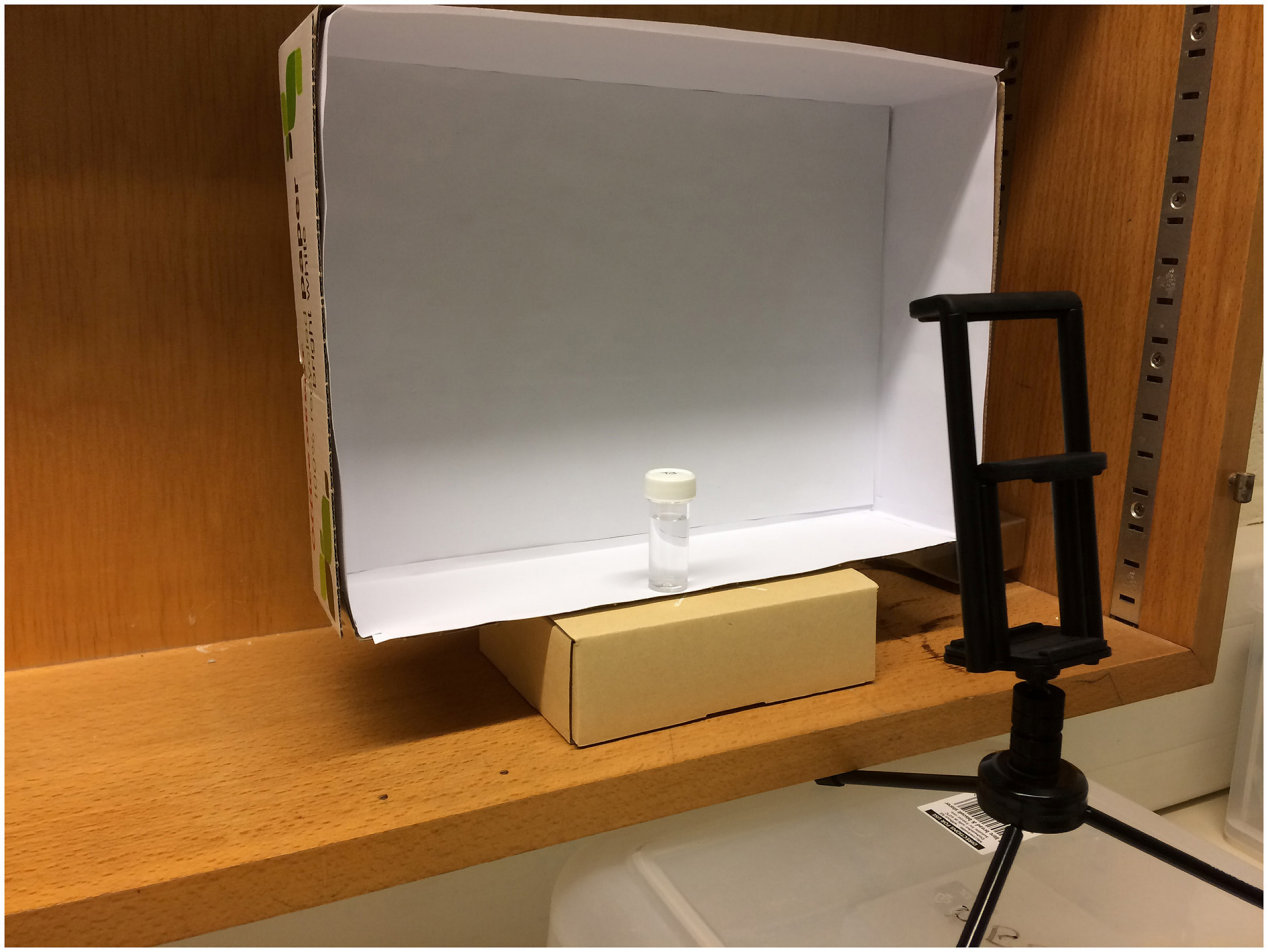
Set up showing the sample and tripod positioning for photographic chlorophyll analysis.

Photographic analysis was conducted by photographing each sample in triplicate (the three photographs were taken immediately one after another without disturbing the sample or camera position) using a smartphone (iPhone 5s, Apple Inc.) digital camera (8 megapixel, 1.5 μm pixels) mounted on a tripod. The camera was positioned at an angle of 30° from vertical at a height of 6.5 cm above the base of the sample flask and a horizontal distance of 8.5 cm from the flask edge; camera position was chosen to center the sample within the photograph and minimize shadowing. All measurements were taken from the center of the camera lens. Samples were shaken vigorously to resuspend any sedimented cells and positioned against a constant white background created by mounting white paper against a card box. The light concentration, measured at the sample position, was ~10 ± 1 μmol photons m^−2^ s^−1^. Figure 1 shows the camera and sample set-up for photographic chlorophyll analysis.

#### RGB Data Analysis

“Green Pixel Intensity” was determined from each photograph using Microsoft Paint software (MS Windows 7, version 6.1). Red, green and blue pixel intensities were obtained using the RGB color model; pixels were selected using the “color picker” tool and the RGB components extracted from the “edit colors” feature.

**Figure 2:**
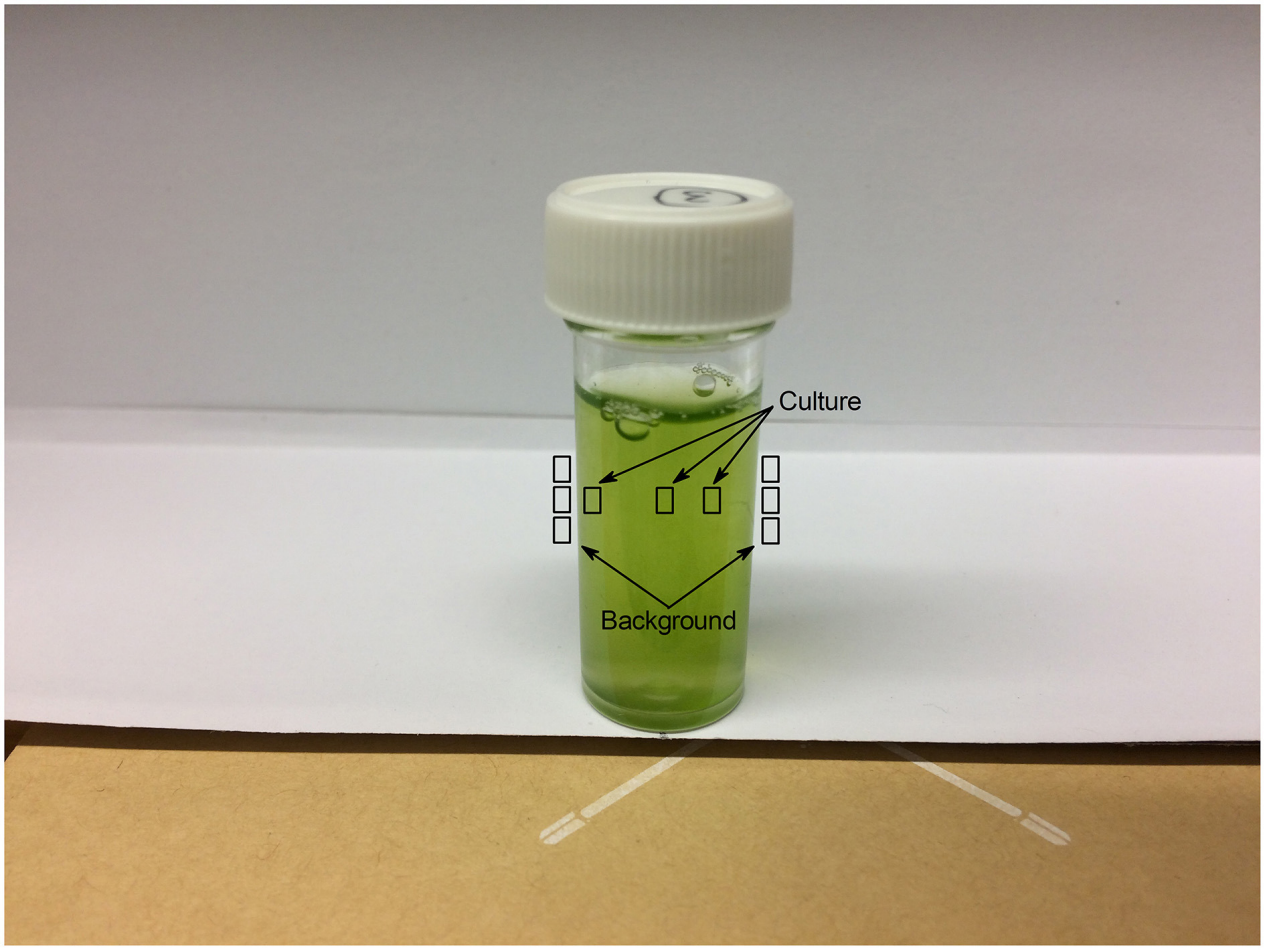
Pixel selection regions within sample photographs for photographic RGB data analysis.

Three individual pixels were selected each from the culture and from the white paper both to the immediate left and right of the flask as a background. Given the shadow present from the flask lid in each image, pixel selections for the culture were made one each from the different shadowed and un-shadowed regions. The sampled regions are shown in Figure 2.

Green pixel intensity (*GPI*) was calculated for each pixel from RGB data according to Equation (1).

(1) $$\begin{array}{l} Green\;Pixel\;Intensity\;\left(GPI\right)=\;\frac{G}{R+G+B} \end{array}$$

where R, G, and B are the red, green and blue pixel intensities, respectively.

For each photograph, *GPI* was calculated for each selected culture and background pixel and the mean *GPI* for each of the culture and background calculated. The mean background *GPI* was subtracted from the mean culture *GPI* such that:

(2) $$\begin{array}{l} GPI_{sample}=GPI_{culture}-GPI_{background} \end{array}$$

where *GPI*_*culture*_ is the mean *GPI* of three pixels selected from the culture and *GPI*_*background*_ is the mean of six pixels taken from the white paper background to the immediate left and right of the culture (Figure 2).

On each day of sampling, a baseline green pixel intensity was obtained as described above from a sample vessel containing medium in the absence of culture and analyzed as above such that:

(3) $$\begin{array}{l} GPI_{baseline}=GPI_{media}-GPI_{background} \end{array}$$

where *GPI*_*media*_ is the mean *GPI* of three pixels selected from the media and *GPI*_*background*_ is the mean of six pixels taken from the white paper background to the immediate left and right of the media (Figure 2).

Once each photograph had been analyzed as above, the mean *GPI*_*sample*_ and mean *GPI*_*baseline*_ were calculated from the corresponding *GPI*_*sample*_ and *GPI*_*baseline*_ calculated values for each of the three photographs taken.

Final green pixel intensity (*GPI*_*final*_) for each sample was obtained by subtracting the mean green pixel intensity of the baseline from that of the sample according to Equation (4).

(4) $$\begin{array}{l} Final\;Green\;Pixel\;Intensity\;\left(GPI_{final}\right)=mean\;GPI_{sample}\\ \;-mean\;GPI_{baseline} \end{array}$$

such that *GPI*_*final*_ represents the mean of the three photographs taken.

### Chlorophyll Analysis by Solvent Extraction

Chlorophyll quantification was conducted spectrophotometrically via solvent extraction in 80% (v/v) acetone/20% (v/v) methanol. A known volume of sample was pelleted in a microcentrifuge (13,000 rpm, ~16,000 g, 10 min) and the supernatant removed; sample volume was selected to maintain an absorbance <1.00 after chlorophyll extraction. The pellet was resuspended in 80% (v/v) acetone in methanol by vortexing before being further centrifuged (13,000 rpm, ~16,000 g, 5 min) to remove cell debris. The absorbance of the supernatant was measured at 646.6, 663.6 and 750 nm in a glass cuvette against an 80% acetone/20% (v/v) methanol blank, using a Jenway 6715 UV/Vis spectrophotometer. Once chlorophyll extraction had taken place, all samples were kept in the dark until analysis to prevent chlorophyll degradation.

Chlorophyll content was calculated according to the extinction coefficients described in Porra et al. (1989) as follows:

(5) $$\begin{array}{l} Chl\;a\;\left(\mu g/ml\right)=\frac{12.25\;E_{663.6}-2.55\;E_{646.6}}{sample\;volume\left(ml\right)} \end{array}$$

(6) $$\begin{array}{l} Chl\;b\;\left(\mu g/ml\right)=\frac{20.31\;E_{646.6}-4.91\;E_{663.6}}{sample\;volume\;\left(ml\right)} \end{array}$$

(7) $$\begin{array}{l} Chl\;a+b\;\left(\mu g/ml\right)=\frac{17.76\;E_{646.6}+7.34\;E_{663.6}}{sample\;volume\;\left(ml\right)} \end{array}$$

where *E*_663.6_ and *E*_646.6_ represent absorbances at 663.6 nm and 646.6 nm minus absorbance at 750 nm, respectively.

### Statistical Analysis

#### Power Analysis

G^*^power (version 3.1) (Faul et al., 2009), was used to conduct a sensitivity power analysis (two tailed; linear bivariate regression: two groups, difference between slopes; α = 0.05; power = 1 – β = 0.80) to determine the detectable effect size (|Δslope|) between each environmental variable and the control dataset for the given sample sizes.

#### General Linear Model Analysis

The relationship between Final Green Pixel Intensity (*GPI*_*final*_), from photographic digital image analysis, and chlorophyll concentration, determined by extraction in 80% (v/v) acetone in methanol, for the control dataset was analyzed by linear regression. Microsoft Excel software was used to calculate the coefficient of determination (*R*^2^) for the correlation assuming a linear relationship between total chlorophyll concentration and green pixel intensity. Data points were subsequently removed (starting with the highest chlorophyll concentration) and the *R*^2^ value recalculated as each additional point was removed. The linear portion of the correlation was chosen as the range of points (>2 points) responsible for the *R*^2^ closest to 1.00. Beyond this point there was also a noticeable increase in the scatter of the data resulting in additional uncertainty in the fit. Regressions for all potential environmental interferences were considered linear within the same region as that of the control dataset.

OriginPro (Origin® 9.1) software was used to compute the 95% confidence band for the linear portion of each curve. The 95% confidence band represents the region in which there is 95% certainty of the true linear fit residing. Analytical characteristics for determination of Chlorophyll *a, b* and total (Chlorophyll *a* and *b*) were determined using methodologies typically used in the development of spectrophotometric methods, for the assessment of key performance indicators including: Limit of Blank (LoB), Limit of Detection (LoD), Linear Interval and Precision, reported as the percent relative standard deviation (%RSD) from the samples used to make the standard curve (Horwitz et al., 1980; Armbruster and Pry, 2008).

IBM® SPSS Statistics® (version 22) was used to compare the slopes and intercepts of different standard curves using a univariate general linear model. A full model was fitted, with different slopes and intercepts for each fitted line. A significant difference between slopes was tested by examining the interaction term; if this was not significant, the interaction term was removed from the model and a significant difference between intercepts was tested. Normality of residuals was checked by visual inspection of Q-Q plots.

## Results

### Validity of the RGB Model for Predicting Chlorophyll Content

To test the validity of the RGB method (Equation 1) to estimate the chlorophyll content of a microalgal culture, a series of sterile 5 ml *C. reinhardtii* samples were made to a range of optical densities in standard Tris-Acetate Phosphate (TAP) Media, pH 7.0–8.5. Samples were first photographed for digital analysis as described in methods before being aliquoted for standard spectroscopic chlorophyll quantification via extraction in 80% (v/v) acetone in methanol.

**Figure 3:**
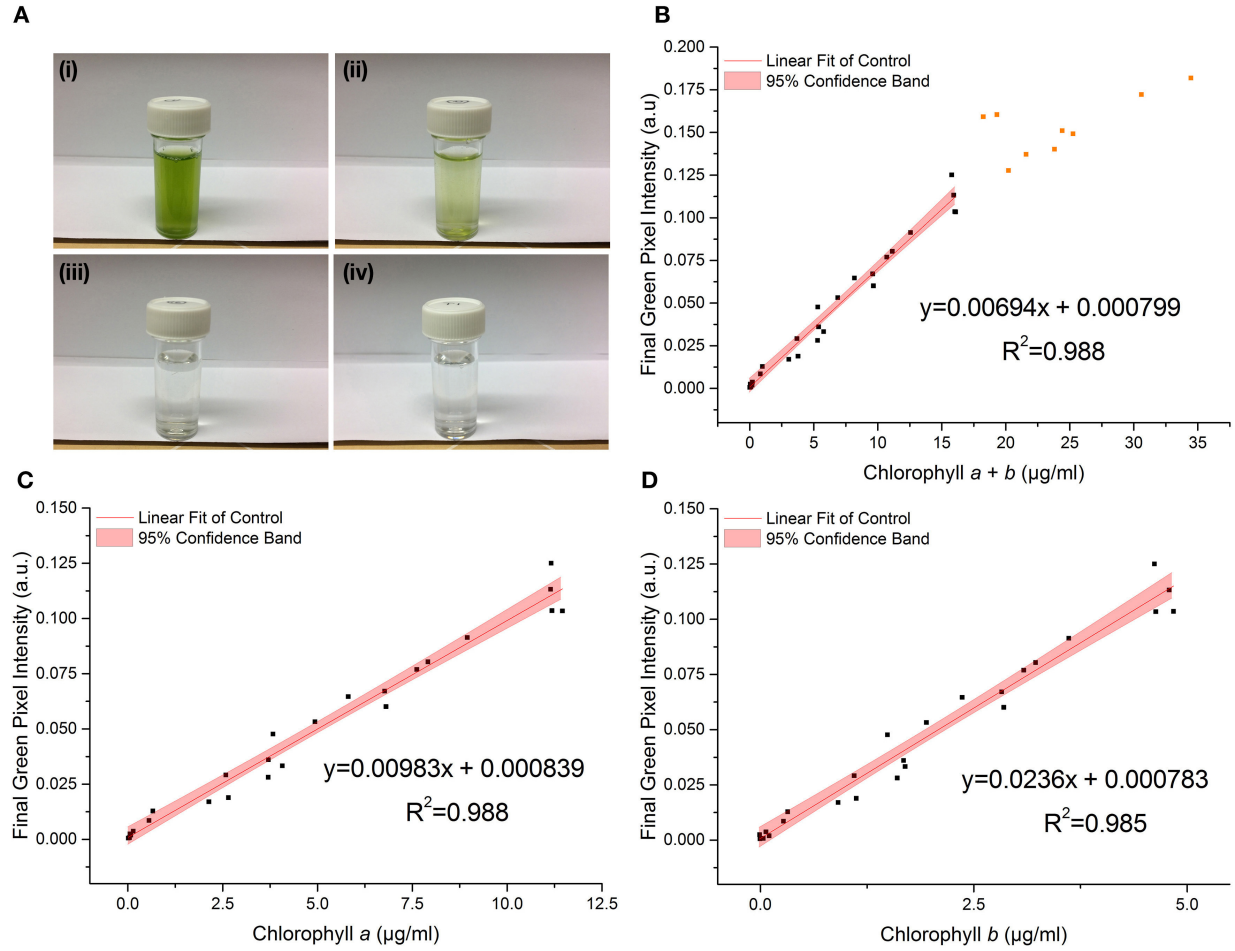
**(A)** Photographs of *C. reinhardtii* samples for RGB digital analysis of chlorophyll concentrations: chlorophyll *a* + *b* = (i) 18.2 μg/ml; (ii) 3.7 μg/ml; (iii) 0.1 μg/ml; (iv) 0 μg/ml (TAP blank). **(B–D)** The correlation between chlorophyll *a* + *b*, chlorophyll *a* and chlorophyll *b* concentrations respectively, as measured by standard extraction method in 80% acetone, and final green pixel intensity calculated from the RGB color model for a sterile culture of wild-type *C. reinhardtii* CC-1690 cultivated in TAP media under 50 μmol photons m^−2^ s^−1^ illumination (16 h photoperiod) and diluted to a range of biomass concentrations for analysis. Data points represent the mean green pixel intensity of three photographs (y) and the mean chlorophyll concentration of three sample aliquots (x). Orange points indicate those excluded from the fitting model due to lack of continued linearity.

Figure 3 shows the relationship between the RGB method and chlorophyll concentration, as measured by the standard extraction method, for the control dataset.

The linear region of the correlation was determined from the correlation between the standard and RGB methods for total chlorophyll (chlorophyll *a* + *b*) concentration (Figure 3B). Adding points individually with increasing chlorophyll concentration resulted in a constant gradient up to a chlorophyll *a* + *b* concentration (*X*) of 16 μg/ml (final green pixel intensity = 0.125). Including points above *X* = 16 μg/ml resulted in the steady reduction of the gradient of the fitted line indicating that the plot tends toward a plateau at high chlorophyll concentrations. In addition, above *X* = 16 μg/ml there is a noticeable increase in the scatter of the plot. The reduction in gradient as well as increased scatter beyond *X* = 16 μg/ml suggests a reduced sensitivity of the RGB method above this point; points above *X* = 16 μg/ml have therefore been excluded from the fitting model and all subsequent plots. For cultures of higher chlorophyll concentration, samples would need to be removed and diluted before analysis. The lower limit for the linear interval was determined by calculating the Limit of Detection (LoD) of the method (Armbruster and Pry, 2008); the resulting LoDs for the determination of total chlorophyll, chlorophyll *a* and chlorophyll *b* were 0.12, 0.10, 0.08 μg/ml, respectively.

There is a strong correlation between the RGB and standard methods for chlorophyll quantification for chlorophyll *a, b* and total chlorophyll as shown by the *R*^2^ values of 0.988, 0.985, and 0.988 respectively. 95% confidence bands demonstrate a high level of precision in the slopes and intercepts of the fitted lines. Data for the control dataset was acquired on three separate days and combined, there was no difference between days in the slopes [*F*_(2, 16)_ = 0.952, *p* = 0.407] or intercepts [*F*_(2, 16)_ = 1.345, *p* = 0.285], thus demonstrating the reproducibility of the method over different sampling periods. The precision of the method was assessed by calculating the percent relative standard deviation (%RSD) from readings included in the standard curve within the linear interval of the method. For chlorophyll quantification for chlorophyll *a, b* and total chlorophyll the average %RSD values were 4.74, 4.98, and 5.75%, respectively.

The high *p*-values for the intercept show that the intercept is not significantly different from zero in each case, which is as expected given the green pixel intensity of the TAP blank has been subtracted from each data point. The regression parameters and analytical characteristics for the control dataset are given in Table 1.

**Table 1:** Regression parameters and analytical characteristics for the control dataset.

	**Equation**	***R*^2^**	***F*_(1,23)_**	***p*^a^**	**LoB, μg/ml**	**LoD, μg/ml**	**Upper limit of linear interval, μg/ml**	**%RSD**
Chl *a* + *b*	*y* = 0.00694(±0.00029) *x* + 0.000799(±0.00198)	0.988	0.162	0.691	0.05	0.12	16.00	5.75
Chl *a*	*y* = 0.00983(±0.00032) *x* + 0.000839(±0.00193)	0.988	0.190	0.667	0.06	0.10	16.00	4.74
Chl *b*	*y* = 0.02361(±0.00086) *x* + 0.000783(±0.00217)	0.985	0.129	0.722	0.05	0.08	16.00	4.98

a *p-values indicating whether the intercept is significantly different from zero in each case*.

*LoB, Limit of Blank; LoD, Limit of Detection; %RSD, Mean Percent Relative Standard Deviation*.

### Effect of Environmental Conditions on the Chlorophyll/RGB Correlation

To investigate whether a single standard curve, for the given experiment, could be used to estimate chlorophyll concentration from green pixel intensity in spite of potential environmental interference, four commonly encountered environmental variables were introduced and their effect on the correlation individually investigated. In each case, linear regression analysis was used to determine whether the slopes and intercepts of each correlation could be considered to be statistically similar to that of the control dataset (Figure 3). Owing to the loss of linearity in the correlation at concentrations above chlorophyll *a* + *b* = 16 μg/ml (green pixel intensity = 0.125), correlations have only been compared up to green pixel intensity = 0.125; data points above this have been removed from the fitted lines.

Table 2 gives the minimum detectable effect size (|Δslope|) for the given sample sizes. The effect size, in this case, is the smallest difference in slope between the RGB/chlorophyll correlations for control and variable datasets that can be distinguished with the given sample sizes.

**Table 2:** Detectable effect size (|Δslope|).

**Sample set vs. control**	**Detectable effect size (|Δslope|)^a^**
Low volume	0.00115
High pH	0.00106
+1.5 ml *E. coli*	0.00107
+0.325 ml *E. coli*	0.00122

a between each measured variable and the control dataset as calculated from G^*^power software; α=0.05; power = 1 – β = 0.80.

**Figure 4:**
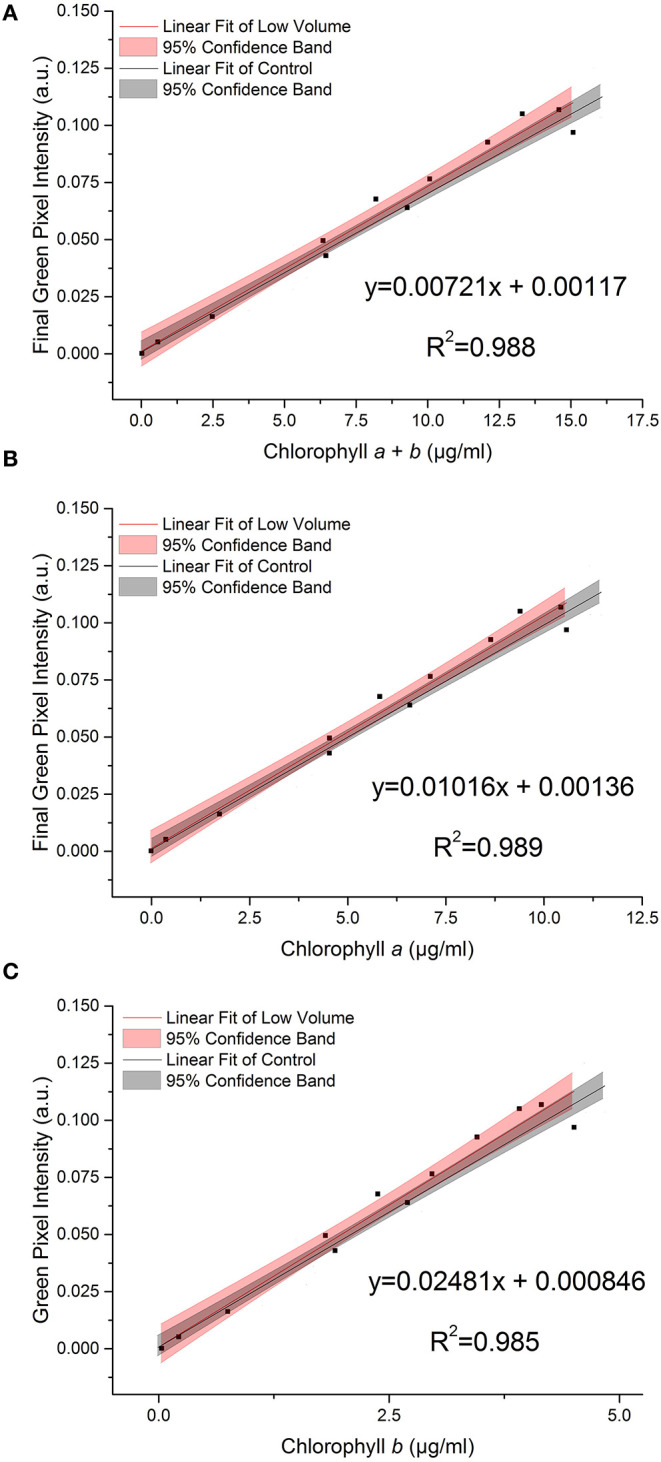
The relationship between final green pixel intensity, calculated from the RGB color method, and **(A)** chlorophyll *a* + *b*, **(B)** chlorophyll *a* and **(C)** chlorophyll *b* concentrations, as measured by standard extraction method in 80% acetone, for a culture of *C. reinhardtii* photographed at two different culture volumes. Red = “low volume”, 3.5 ml samples; Gray = control, 5 ml samples. Data points represent the mean green pixel intensity of three photographs (y) and the mean chlorophyll concentration of three sample aliquots (x).

Figure 4 shows the effect on the relationship between green pixel intensity and chlorophyll *a, b* and total chlorophyll when the photographed sample volume is reduced from 5.0 to 3.5 ml. Samples were photographed at a volume of 3.5 ml for comparison with the control samples (5 ml) before being analyzed for chlorophyll concentration by extraction in 80% (v/v) acetone in methanol. In each case the linear fit for the low volume samples (red) is shown next to that of the control dataset (gray) with the 95% confidence bands of each fit. In each case the *R*^2^ values of 0.988, 0.989 and 0.985 for total chlorophyll (*a* + *b*), chlorophyll *a* and chlorophyll *b*, respectively demonstrate a strong linear relationship between the green pixel intensity and chlorophyll concentration for the lower sample volume.

Comparing the low volume and control datasets, we found no significant difference between the slopes of the two lines (*p* > 0.05, Table 3); this is corroborated by the overlapping 95% confidence bands in each case.

**Table 3:** Regression parameters for “Low Volume” samples.

			**Slope^a^**		**Intercept^b^**	
	**Equation**	***R*^2^**	***F*_(1, 33)_**	***p***	***F*_(1, 34)_**	***p***
Chl *a + b*	*y* = 0.00721(±0.00035) *x* + 0.00117(±0.00337)	0.988	0.405	0.529	1.292	0.264
Chl *a*	*y* = 0.01016(±0.00047) *x* + 0.00136(±0.00320)	0.989	0.335	0.567	1.251	0.271
Chl *b*	*y* = 0.02481(±0.00139) *x* + 0.00085(±0.00388)	0.985	0.545	0.466	1.328	0.257

a *p-values indicating whether the slope of the fitted line is significantly different from that of the control dataset in each case*.

b *p-values indicating whether the intercept of the fitted line is significantly different from zero and from the control dataset in each case*.

Similarly, we found no significant difference between the intercepts of the low volume and control datasets (*p* > 0.05, Table 3).

**Figure 5:**
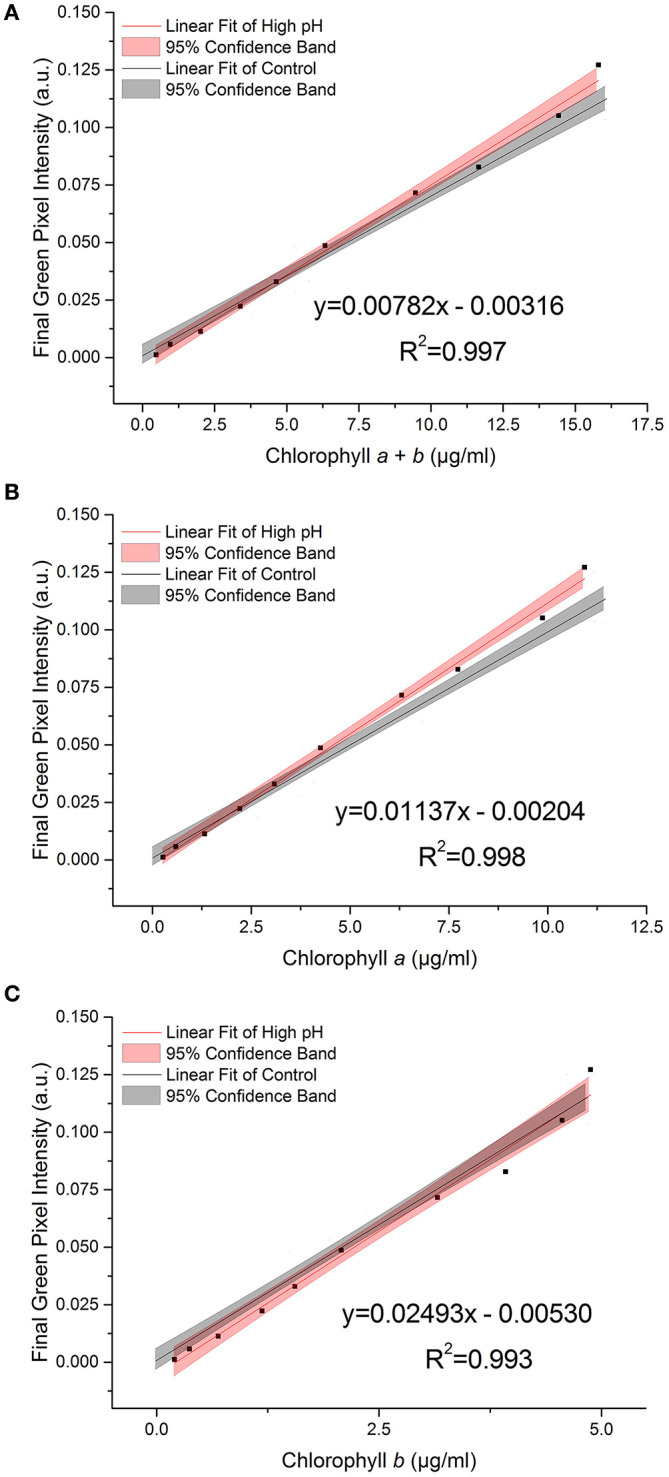
The relationship between final green pixel intensity, calculated from the RGB color method, and **(A)** chlorophyll *a* + *b*, **(B)** chlorophyll *a* and **(C)** chlorophyll *b* concentrations, as measured by standard extraction method in 80% acetone, for a culture of *C. reinhardtii* photographed at two different culture pHs. Red = “high pH”, pH 9.5 samples; Gray = control, pH 7.0–8.5 samples. Data points represent the mean green pixel intensity of three photographs (y) and the mean chlorophyll concentration of three sample aliquots (x).

During growth, even buffered cultures can vary in their pH owing to the consumption and release of carbon dioxide during photosynthesis and respiration. A selection of samples were transferred into high pH media (pH 9.5) immediately before being photographed in order to test the effect of pH on the RGB method. Figure 5 shows the relationship between green pixel intensity and conventional chlorophyll quantification for high pH samples compared to the control dataset (7.0 < pH < 8.5). The control dataset is present at a range of pH values owing to the increase in pH as the culture grows. The strong linear relationship is maintained despite the increase in pH as is evident from the high *R*^2^ values (*R*^2^ > 0.995 in each case).

We found a significant difference between the slopes of the high pH and control data for total chlorophyll and chlorophyll *a* (*p* = 0.035 and 0.009 respectively, Table 4). In contrast, for chlorophyll *b* there is no significant difference in the slope or intercept compared with that of the control data (*p* = 0.383) indicating that chlorophyll *b* concentration determined from the RGB color method is less sensitive to change in the culture pH. This can be seen clearly from the plots of the correlations against the control correlation (Figure 5). With the exception of chlorophyll *b*, the high pH correlation is steeper than the corresponding correlation for the control dataset.

**Table 4:** Regression parameters for “High pH” samples.

			**Slope^a^**		**Intercept^b^**	
	**Equation**	***R*^2^**	***F*_(1, 31)_**	***p***	***F*_**(1, 32)**_**	***p***
Chl *a + b*	*y* = 0.00782(±0.00021) *x* – 0.00316(±0.00186)	0.997	4.868	0.035	–	–
Chl *a*	*y* = 0.01137(±0.00026) *x* – 0.00204(±0.00154)	0.998	7.890	0.009	–	–
Chl *b*	*y* = 0.02493(±0.00104) *x* – 0.00530(±0.00292)	0.993	0.783	0.383	1.825	0.186

a *p-values indicating whether the slope of the fitted line is significantly different from that of the control dataset in each case*.

b *p-values indicating whether the intercept of the fitted line is significantly different from zero and from the control dataset in each case*.

**Figure 6:**
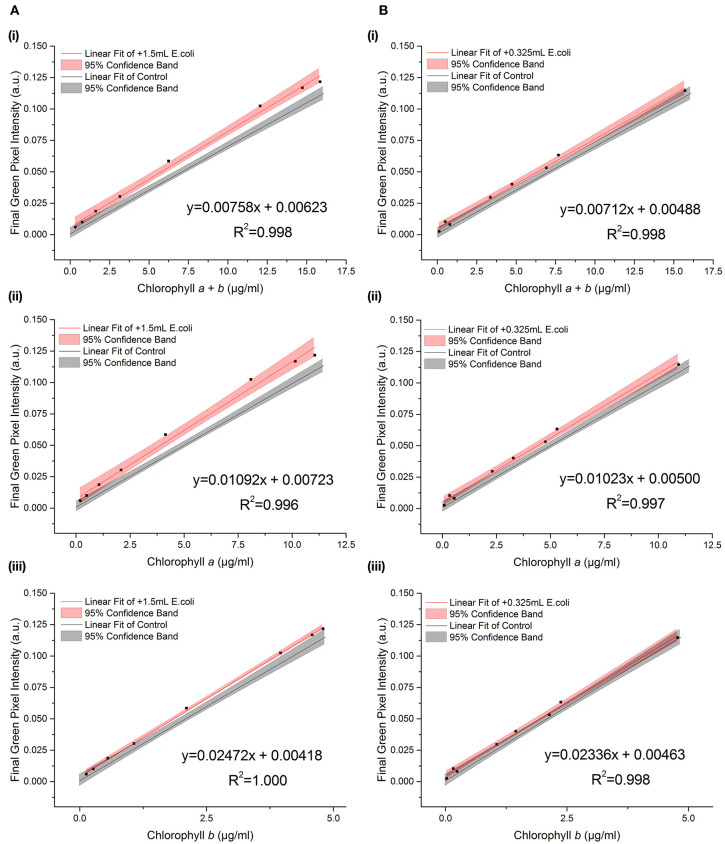
The relationship between final green pixel intensity, calculated from the RGB color method, and (i) chlorophyll *a* + *b*, (ii) chlorophyll *a* and (iii) chlorophyll *b* concentrations, as measured by standard extraction method in 80% acetone, for a culture of *C. reinhardtii* contaminated with two different concentrations of *E. coli*. **(A)**: Red = addition of 1.5 ml E. coli (A_600_ ≈ 1.0); Gray = control, sterile samples. **(B)**: Red = addition of 0.325 ml E. coli (A_600_ ≈ 0.25); Gray = control, sterile samples. Data points represent the mean green pixel intensity of three photographs (y) and the mean chlorophyll concentration of three sample aliquots (x).

*E. coli* was added at two different concentrations (A_600_ ≈ 1.0 and 0.25) to a range of *C. reinhardtii* samples to investigate the effect of bacterial contamination on the RGB method. Figure 6 shows the relationship between the two methods for each concentration of *E. coli* (red) compared to the control dataset (gray). The clear linear relationship between the two methods is maintained despite the bacterial contamination (*R*^2^ > 0.995 in both cases). There was no significant difference between the slopes of the contaminated samples and control data for chlorophyll *a, b* or total chlorophyll concentration (Table 5).

**Table 5:** Regression parameters for contaminated samples.

				**Slope^a^**		**Intercept^b^**	
		**Equation**	***R*^2^**	***F*_(1, 29)_**	***p***	***F*_**(1, 30)**_**	***p***
+1.5 ml *E. coli*	Chl *a + b*	*y* = 0.00758(±0.00022) *x* + 0.00623(±0.00198)	0.998	2.526	0.123	16.156	<0.001
	Chl *a*	*y* = 0.01092(±0.00041) *x* + 0.00723(±0.00257)	0.996	3.542	0.070	21.394	<0.001
	Chl *b*	*y* = 0.02472(±0.00025) *x* + 0.00418(±0.00071)	1.000	0.623	0.436	5.301	0.028
+0.325 ml *E. coli*	Chl *a + b*	*y* = 0.00712(±0.00019) *x* + 0.00488(±0.00132)	0.998	0.142	0.709	4.704	0.038
	Chl *a*	*y* = 0.01023(±0.00029) *x* + 0.00500(±0.00139)	0.997	0.395	0.535	6.233	0.018
	Chl *b*	*y* = 0.02336(±0.00056) *x* + 0.00463(±0.00118)	0.998	0.022	0.882	1.894	0.179

a *p-values indicating whether the slope of the fitted line is significantly different from that of the control dataset in each case*.

b *p-values indicating whether the intercept of the fitted line is significantly different from zero and from the control dataset in each case*.

However, when fitting two lines with the same slope, there was a significant difference between the intercepts of the contaminated and control datasets for both the high and low *E. coli* cases, except for low *E. coli* chlorophyll *b* (Table 5). This difference in the intercepts of the contaminated and control correlations can be clearly seen in Figure 6, indicating that the high *E. coli* contamination is causing a zero error in the intercept affecting the detection limit of the method. That can be easily corrected if the level of contamination is known.

**Figure 7:**
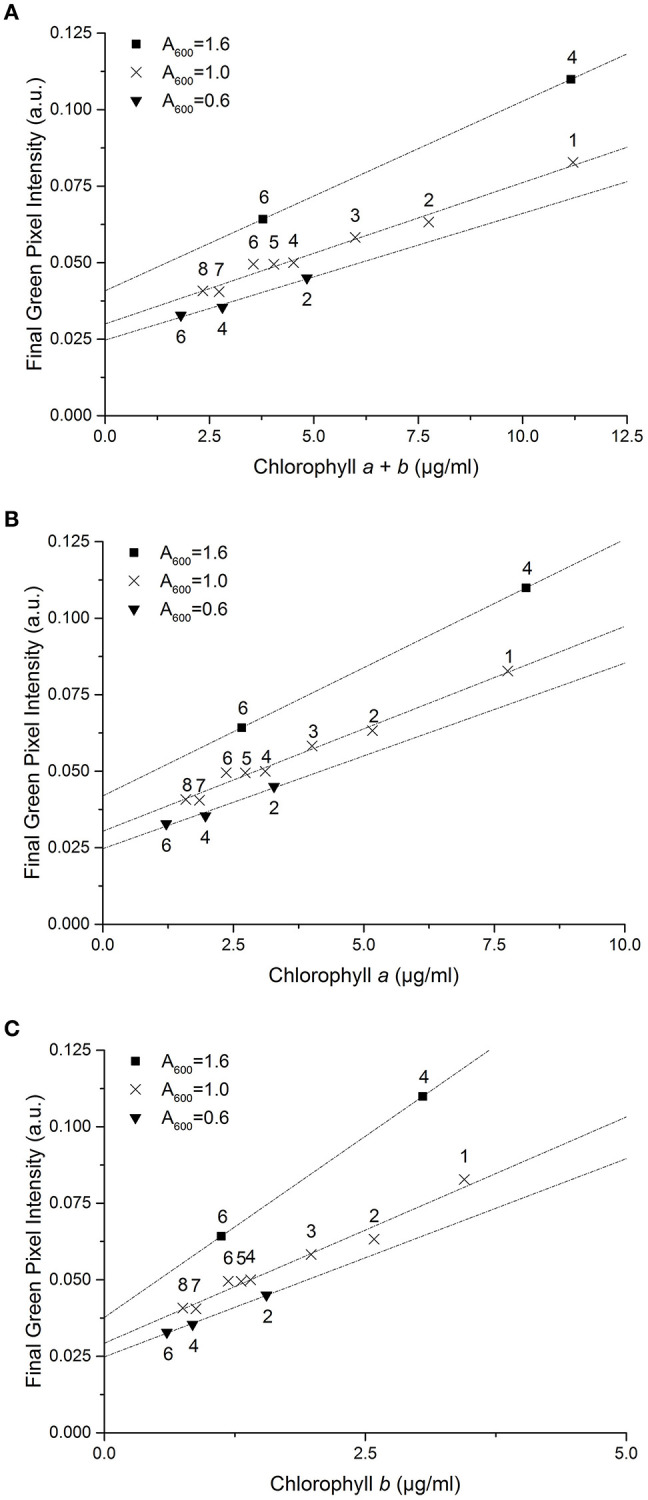
The correlation between final green pixel intensity, calculated from the RGB color method, and **(A)** chlorophyll *a* + *b*, **(B)** chlorophyll *a* and (**C**) chlorophyll *b* concentrations, as measured by standard extraction method in 80% acetone, for a culture of *C. reinhardtii* in TAP-N media and photographed over 8 days with three initial starting biomass concentrations as indicated by the different A_600_ values. Numbers above data points represent the number of days after inoculation that analysis took place. Data points represent the mean green pixel intensity of three photographs (y) and the mean chlorophyll concentration of three sample aliquots (x) and lines represent linear fits through the points.

When starved of nitrogen, microalgae typically accumulate neutral lipids and cellular chlorophyll content is seen to deplete; this is proposed to be due to recycling of the chloroplast membrane lipids in favor of neutral lipid accumulation as an energy store (Moellering and Benning, 2010; Valledor et al., 2014). Reduction in chlorophyll concentration therefore has the potential to be used in many cases as an early indicator of neutral lipid accumulation. To test the effect of this process on the RGB method, a series of samples at a three optical densities (A_600_ = 1.6 – 0.6) were transferred into TAP-N media and analyzed periodically, one sample per starting optical density over 8 days, as the chlorophyll content gradually decreased. Figure 7 shows the effect of nitrogen starvation on the correlation between green pixel intensity and chlorophyll concentration; individual symbols represent different starting optical densities.

Decreasing initial culture absorbance (A_600_) is shown to result in a decrease in the measured green pixel intensity at similar chlorophyll concentrations. Similarly, the green pixel intensity at zero chlorophyll concentration is shown to be dependent upon the initial optical density of the samples. For nitrogen starved *C. reinhardtii* there is a clear difference in the correlations between methods compared with that of the control dataset.

Table 6 gives the error generated, as a percentage of the actual value, if the standard curve for the control dataset is used to estimate chlorophyll concentration for each environmental variable; TAP-N correlations are not included owing to the poor similarity with the control. In each case, the green pixel intensity corresponding to the maximum and half maximum measurable chlorophyll concentrations for the control dataset are used to calculate the predicted and actual chlorophyll concentrations for each variable in order to calculate the generated errors.

**Table 6:** Percentage error generated when using control data standard curve to estimate chlorophyll concentration in the presence of environmental interference^*^.

	**Total chlorophyll**		**Chl *a***		**Chl *b***	
	**% error at max**	**% error at half max**	**% error at max**	**% error at half max**	**% error at max**	**% error at half max**
Low volume	4.3	4.6	3.9	4.4	5.1	5.2
High pH	8.8	5.2	12.9	10.2	0.1	−4.8
+1.5 ml *E. coli*	14.9	21.1	17.8	25.3	7.9	11.4
+0.325 ml *E. coli*	6.4	10.7	8.1	12.4	2.4	6.2

* *Percentages are calculated at the maximum measurable and half maximum measurable chlorophyll concentration from the control data standard curve*.

For the low volume, high pH and low *E. coli* concentration the errors are typically ≤ 10% indicating only a relatively small overall error in the estimate of chlorophyll concentration. The standard curve for the control dataset could be used in each of these cases with relatively small errors. In contrast, there is a large error introduced when there is larger *E. coli* contamination, with errors typically between 15 and 25%. Use of the standard curve of the control dataset would yield greater errors when high levels of contamination are present. The errors shown also corroborate that *E. coli* contamination induces a zero error in the intercept of the standard curve as can be seen from the increased percentage error at lower chlorophyll concentrations, while high pH results in an increased slope of the fit, seen from the reduced error at lower total chlorophyll and chlorophyll *a* concentrations.

## Discussion

This study presents a simple and non-destructive method by which the concentration of chlorophyll *a, b* and total chlorophyll of a microalgal culture can be estimated from the green pixel intensity of digital photographs using a standard calibration curve. The ability to determine chlorophyll *in situ* avoids repeated sampling, which is a problem with small culture volumes where there is insufficient material for repeated removal of sample, and which also risks introducing contamination. Compared with other digital image analysis methods (Su et al., 2008; Dey et al., 2016; Friedman et al., 2016), the method reported here needs only simple, easily available digital camera equipment and software and no complicated analysis. It should be easy to implement in different laboratory settings once a standard curve has been established for the specific experimental set up.

There is a strong linear relationship (*R*^2^ ≥ 0.985) between green pixel intensity, calculated from the tested method, and total chlorophyll concentration, as measured by a standard spectroscopic method with chlorophyll extraction in 80% (v/v) acetone/20% methanol (Porra et al., 1989) for a sterile culture of *C. reinhardtii* in TAP media (pH 7.0–8.5), photographed at a sample volume of 5 ml, up to a concentration of 16 μg/ml total chlorophyll (green pixel intensity = 0.125). Above this, the gradient of the slope decreases indicating the curve tends toward a plateau at high chlorophyll concentration. From this, the limit of sensitivity for the method has been estimated at a green pixel intensity of 0.125 and therefore samples with higher chlorophyll concentration (>16 μg/ml) should be diluted to fall within the linear interval of the RGB method.

A selection of commonly encountered environmental variables were chosen to investigate the sensitivity of the method to environmental interference. For each variable investigated, the excellent linear relationship between green pixel intensity and chlorophyll concentration was maintained.

Statistical analysis revealed no significant difference in the green pixel intensity/chlorophyll correlation when the photographed sample volume was decreased. This indicates that the method is insensitive to this factor and a single standard curve created could be used to estimate chlorophyll concentration. This is particularly important for time-course studies where the culture volume may be reduced gradually over time as a result of other analyses.

**Figure 8:**
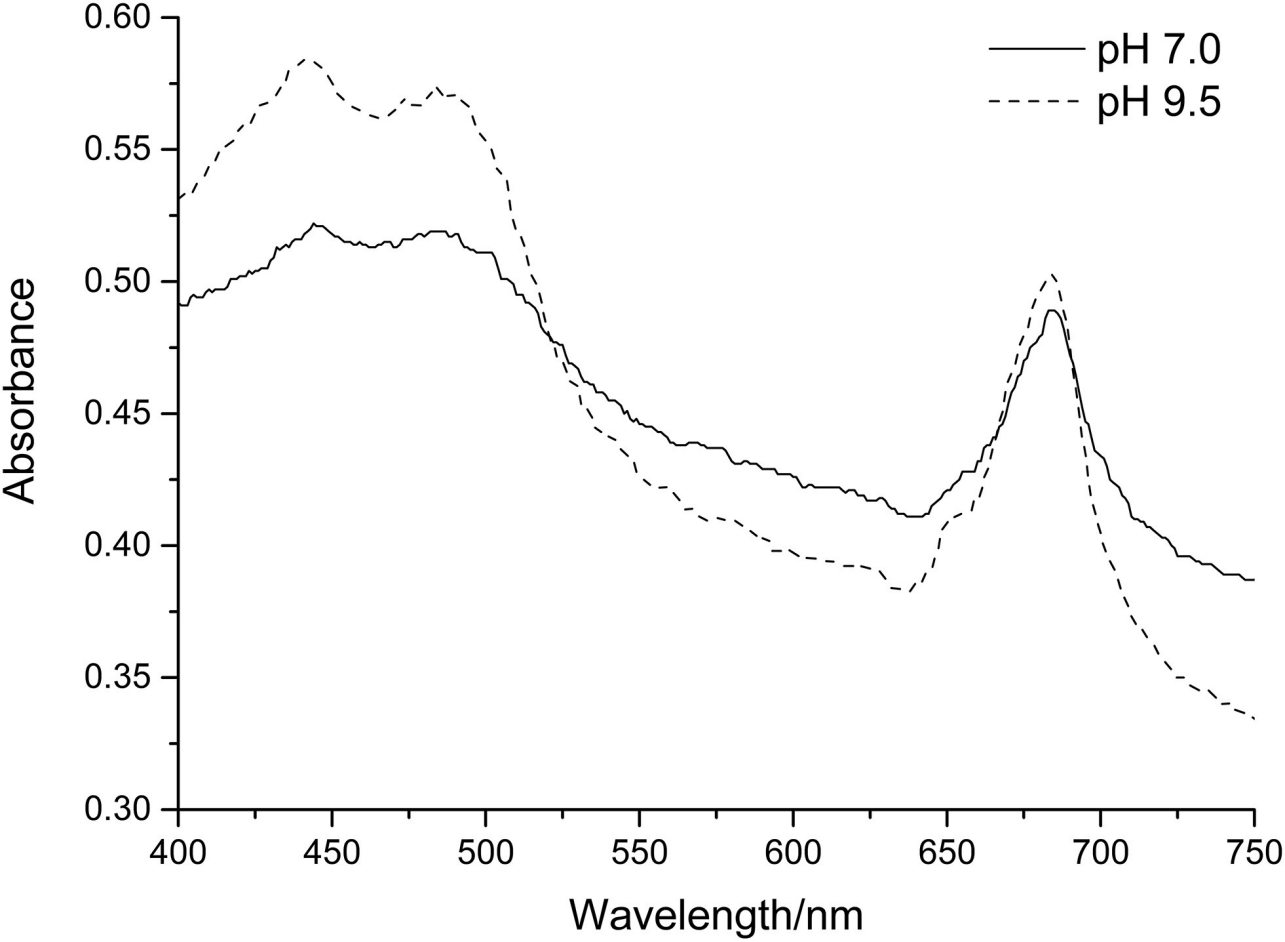
Absorption spectra of *C. reinhardtii* in Tris-Acetate-Phosphate (pH 7.0) and CAPS-Acetate-Phosphate (pH 9.5) media. In each case spectra have been normalized to a chlorophyll *a* + *b* concentration = 7.5 μg/ml.

In contrast, we found that there is a statistically significant increase in the slope of the green pixel intensity/chlorophyll correlation for both total chlorophyll and chlorophyll *a* when pH is increased. Figure 8 shows the visible range absorbance spectra for a culture of *C. reinhardtii* at pH 7.0 and 9.5. For the sake of comparison, the pH 9.5 spectrum has been adjusted to a total chlorophyll concentration equivalent to that of the pH 7.0 sample, assuming a directly proportional relationship between total chlorophyll concentration and biomass optical density. As can be seen from the spectra, the higher pH sample has a lower absorbance in the green region of the spectrum (~520–560 nm) and higher absorbance in both the red (~630–750 nm) and blue (~450–490 nm) regions of the spectrum. These differences will result in the culture presenting with a stronger green color and are likely responsible for the increased green pixel intensity of the high pH samples at similar chlorophyll concentrations given that green pixel intensity is defined as the ratio of the green pixel component over the sum of red, green and blue components (Equation 1). This increase in green color at high pH may be as a result of chlorophyll conversion to chlorophyllin which occurs via the removal of a hydrocarbon side chain and replacement of the central magnesium ion with copper; copper chlorophyllin has a much more intense green color compared with non-copper chlorophyll (Kendrick, 2012).

Addition of *E. coli* (A_600_ ≈ 1.0) revealed a significant increase in the intercept of the green pixel intensity/chlorophyll regression for total chlorophyll and chlorophyll *a*, as can be seen from Figure 6. It is likely that a zero error in the intercept is being induced by the opacity of *E. coli* even in the absence of algal biomass. The magnitude of this error was, as expected, higher for high levels of contamination. Given that there is no significant difference in the slope of the line with *E. coli* contamination, the zero error in the intercept could likely be corrected for where the level of contamination is known.

These observed differences in the lines for the *E. coli* and high pH cases were much lower, or absent, for chlorophyll *b* than total chlorophyll and chlorophyll *a*. The reason for this is not understood but indicates chlorophyll *b*, as calculated from the RGB method, is less sensitive to changing environmental conditions. The control correlation could be used as a standard curve to estimate chlorophyll *b* concentration from green pixel intensity despite the environmental interferences investigated.

In contrast to the other environmental interferences investigated, there is a very clear difference between the correlations for nitrogen starved samples compared with that of the control dataset. For each initial biomass concentration, the slope of the correlation is much shallower than that of the control dataset and there is a clear increase in the intercept of the correlation with increasing initial biomass concentration. Both these factors would result in a significant overestimation of the chlorophyll concentration from the green pixel intensity if the original standard curve is used. We propose that this is most likely due to the yellow color of the lipids as chlorophyll concentration decreases. Given the proximity of yellow and green within the visible spectrum, the residual yellow color will likely cause significant interference with the green pixel intensity. The green component of the RGB scale has previously been shown to be significant when estimating the lipid content of a microalgal culture using digital image analysis (Su et al., 2008). Without a significant correction for lipid accumulation, the developed method is not suitable for estimating chlorophyll concentration when chlorophyll concentration is reduced as a result of nitrogen starvation due to interference from neutral lipid accumulation. Despite this, the method could be applied to qualitatively determine if chlorophyll concentration is increasing/decreasing.

This method extends other digital analysis methods by investigating its applicability over a wide range of commonly encountered environmental variables. The insensitivity of this method to small changes in sample volume and low concentrations of bacterial contamination means that the method could be used with a single standard curve in spite of these changes, making this method useful for a range of experimental investigations. The method is also shown to be useful when high levels of bacterial contamination are present or with a variable culture pH, however these conditions may lead to higher errors in the estimation of total chlorophyll and chlorophyll *a*. In addition, the method is unique in its minimal use of data processing all of which can be conducted through widely accessible software packages.

## Conclusions

Digital image analysis has been used to develop an inexpensive and rapid method to estimate chlorophyll *a, b* and total chlorophyll concentration of an algal culture without sample destruction. Comparison of the Green Pixel Intensity (GPI) method described and a standard spectroscopic method for chlorophyll quantification of a culture of *C. reinhardtii* CC-1690 revealed a strong linear correlation (*R*^2^ > 0.985) up to a green pixel intensity of 0.125, corresponding to a total chlorophyll concentration of 16 μg/ml in this case. The standard curve created is robust despite changes in sample volume and small quantities of bacterial contamination and can therefore be used in these cases without modification. Increasing pH resulted in a small increase in the slope of the GPI/chlorophyll correlation but errors in chlorophyll estimation remained small. In contrast, large quantities of bacterial contamination result in an error in the intercept of the standard curve leading to overestimations of chlorophyll concentration. It is likely that correction factors could be found and applied where the level of contamination is known. Lipid accumulation, as a result of nitrogen deprivation, resulted in significant changes to the GPI/chlorophyll correlation proposed to be due to yellowing of the culture as chlorophyll was depleted and neutral lipids accumulated. As such the method, as it stands, is not appropriate for cultures with reduced chlorophyll concentrations as a result of significant lipid accumulation.

This method has the potential to be applied widely to different algal culture situations or even environmental samples, particularly in situations where sample and equipment availability may be limited. This could include, for example, field laboratories and laboratories in developing countries, school laboratories or citizen science projects. It would require following the methodology described to construct a standard curve relating chlorophyll content to GPI for the specific algal species, culture vessel and photographic set up. Although the experiments presented here used an iPhone 5S and Microsoft Paint, in principle any digital camera and software capable of analyzing RGB pixel intensity could be used as long as the conditions used for establishing the standard curve (growth, photographic set up, and software) are subsequently replicated precisely for the experimental samples. To the best of our knowledge, the simplicity and accessibility of this method is unique compared with other non-invasive chlorophyll quantification methods, requiring very little equipment, expertise or specialist software.

## Data Availability Statement

All datasets generated for this study are included in the article/Supplementary Material.

## Author Contributions

NW contributed to the conception and design of experimental work, collection, assembly and analysis of the data and produced the article draft. AB and MC-V contributed to the conception and design of experimental work and critically revised the article. RQ contributed to the statistical analysis and interpretation of the data, and critically revised the article. All authors read and approved the submitted manuscript.

## Conflict of Interest

The authors declare that the research was conducted in the absence of any commercial or financial relationships that could be construed as a potential conflict of interest.

## References

[B1] Armbruster D. A. Pry T. (2008). Limit of blank, limit of detection and limit of quantitation. Clin. Biochem. Rev. 29, S49–S52. 18852857 PMC2556583

[B2] Buschmann C. Langsdorf G. Lichtenthaler H. (2000). Imaging of the blue, green, and red fluorescence emission of plants: an overview. Photosynthetica 38, 483–491. 10.1023/A:1012440903014

[B3] Chisti Y. (2007). Biodiesel from microalgae. Biotechnol. Adv. 25, 294–306. 10.1016/j.biotechadv.2007.02.001 17350212

[B4] Chlamydomonas Resource Center. (2016). TAP and Tris-minimal (online). Available online at: https://www.chlamycollection.org/methods/media-recipes/tap-and-tris-minimal/ (accessed November 10, 2016).

[B5] Dey A. K. Sharma M. Meshram M. R. (2016). An analysis of leaf chlorophyll measurement method using chlorophyll meter and image processing technique. Proc. Comput. Sci. 85, 286–292. 10.1016/j.procs.2016.05.235

[B6] Faul F. Erdfelder E. Buchner A. Lang A.-G. (2009). Statistical power analyses using G^*^Power 3.1: tests for correlation and regression analyses. Behav. Res. Methods 41, 1149–1160. 10.3758/BRM.41.4.1149 19897823

[B7] Friedman J. M. Hunt E. R. Jr Mutters R. G. (2016). Assessment of leaf color chart observations for estimating maize chlorophyll content by analysis of digital photographs. Agron. J. 108, 822–829. 10.2134/agronj2015.0258

[B8] Goncalves E. C. Wilkie A. C. Kirst M. Rathinasabapathi B. (2016). Metabolic regulation of triacylglycerol accumulation in the green algae: identification of potential targets for engineering to improve oil yield. Plant Biotechnol. J. 14, 1649–1660. 10.1111/pbi.12523 26801206 PMC5066758

[B9] Gorman D. S. Levine R. P. (1965). Cytochrome F and plastocyanin: their sequence in the photosynthetic electron transport chain of *Chlamydomonas reinhardi*. Proc. Natl. Acad. Sci. U.S.A. 54, 1665–1669. 10.1073/pnas.54.6.1665 4379719 PMC300531

[B10] Gupta S. D. Ibaraki Y. Pattanayak A. K. (2013). Development of a digital image analysis methods for real-time estimation of chlorophyll content in micropropagated potato plants. Plant Biotechnol. Rep. 7, 91–97. 10.1007/s11816-012-0240-5

[B11] Horwitz W. Kamps L. R. Boyer K. W. (1980). Quality assurance in the analysis of foods and trace constituents. J. Assoc. Off. Anal. Chem. 63, 1344–1354. 10.1093/jaoac/63.6.1344 7451398

[B12] Kendrick A. (2012). Natural Food Additives, Ingredients and Flavourings: 2 – Natural Food and Beverage Colourings. Cambridge, UK: Woodhead Publishing Series in Food Science, Technology and Nutrition, 24–40. 10.1533/9780857095725.1.25

[B13] Liu B. Benning C. (2013). Lipid metabolism in microalgae distinguishes itself. Curr. Opin. Biotechnol. 24, 300–309. 10.1016/j.copbio.2012.08.008 22981869

[B14] Moellering E. R. Benning C. (2010). RNA interference silencing of a major lipid droplet protein affects lipid droplet size in *Chlamydomonas reinhardtii*. Eukaryot. Cell 9, 97–106. 10.1128/EC.00203-09 19915074 PMC2805299

[B15] Netto A. T. Campostrini E. de Oliveira J. G. Bressan-Smith R. E. (2005). Photosynthetic pigments, nitrogen, chlorophyll a fluorescence and SPAD-502 readings in coffee leaves. Sci. Hortic. 104, 199–209. 10.1016/j.scienta.2004.08.013

[B16] Porra R. J. Thompson W. A. Kriedemann P. E. (1989). Determination of accurate extinction coefficients and simultaneous equations for assaying chlorophylls a and extracted with four different solvents: verification of the concentration of chlorophyll standards by atomic absorption spectroscopy. Biochem. Biophys. Acta 975, 384–394. 10.1016/S0005-2728(89)80347-0

[B17] Rignon J. P. G. Capuani S. Fernandes D. M. Guimarães T. M. (2016). A novel method for the estimation of soybean chlorophyll content using a smartphone and image analysis. Photosynthetica 54, 559–566. 10.1007/s11099-016-0214-x

[B18] Siaut M. Cuiné S. Cagnon C. Fessler B. Nguyen M. Carrier P.. (2011). Oil accumulation in the model green alga *Chlamydomonas reinhardtii*: characterisation, variability between common laboratory strains and relationship with starch reserves. BMC Biotechnol. 11:7. 10.1186/1472-6750-11-7 21255402 PMC3036615

[B19] Su C.-H. Fu C.-C. Chang Y.-C. Nair G. R. Ye J.-L. Chu I.-M.. (2008). Simultaneous estimation of chorophyll a and lipid contents in microalgae by three-color analysis. Biotechnol. Bioeng. 99, 1034–1039. 10.1002/bit.21623 17705233

[B20] Valledor L. Furuhashi T. Recuenco-Muñoz L. Wienkoop S. Weckwerth W. (2014). System-level network analysis of nitrogen starvation and recovery in *Chlamydomonas reinhardtii* reveals potential new targets for increased lipid accumulation. Biotechnol. Biofuels 7, 171–187. 10.1186/s13068-014-0171-1 25663847 PMC4320484

[B21] Vincent W. F. (1983). Fluorescence properties of the freshwater phytoplankton: three algal classes compared. Br. Phycol. J. 18, 5–21. 10.1080/00071618300650021

[B22] Wang H. Zhu R. Zhang J. Ni L. Shen H. Xie P. (2018). A novel and convenient method for early warning of algal cell density by chlorophyll fluorescence parameters and its application in a highland lake. Front. Plant Sci. 9:869. 10.3389/fpls.2018.00869 30002664 PMC6031977

[B23] Wijffels R. H. Barbosa M. J. (2010). An outlook on microalgal biofuels. Science 329, 796–799. 10.1126/science.1189003 20705853

[B24] Wood A. M. Everroad R. C. Wingard L. M. (2005). Chapter 18: Measuring growth rates in microalgal cultures, in Algal Culturing Techniques, ed Andersen R. A. (Burlington, MA; San Diego, CA; London, UK: Elsevier Academic Press) 269–286.

