# Supplementary material for: A Simple and Non-destructive Method for Chlorophyll Quantification of Chlamydomonas Cultures Using Digital Image Analysis
Source: Front Bioeng Biotechnol. 2020 Jul 21;8:746. doi: 10.3389/fbioe.2020.00746 (PMC7386287; doi:10.3389/fbioe.2020.00746)
Supplement: Supplementary file 1 [file Presentation_1.pdf]

Appendix 1 – Protocol

The following protocol details the recommended method to create a standard curve with which to estimate chlorophyll concentration of an algal culture from green pixel intensity obtained through digital image analysis.

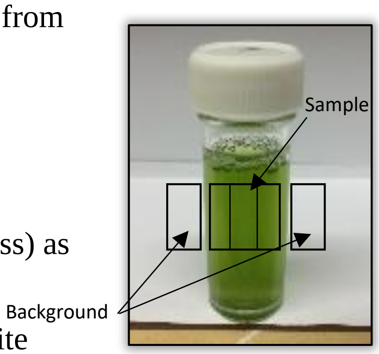

1. Grow a culture of your organism to stationary phase

2. Dilute the culture to a range of optical densities/biomass concentrations into the culture vessels you will use for the experiment. Include a sample containing only media (no biomass) as a blank.

3. Take photographs of each sample (in triplicate) against a white background (this can be obtained by mounting white paper against a card backdrop) at a constant camera position. Choose a location that will not be affected significantly by changes in light – e.g. a room with no windows.

4. Take three aliquots from each sample flask and use to measure chlorophyll by a standard analytical method.

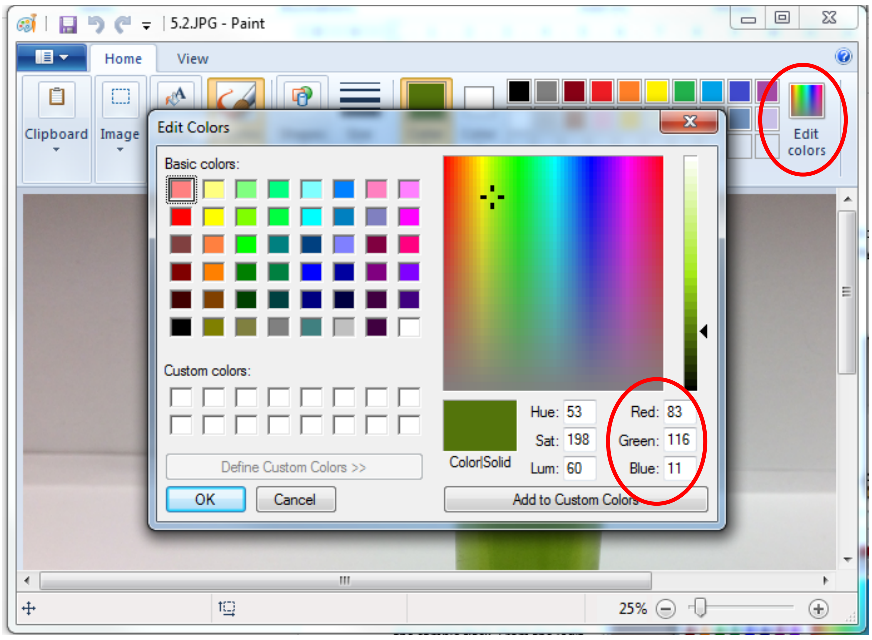

5. Load photographs into Microsoft Paint software and using the ‘eyedropper’ tool select a pixel from the left of the sample flask. From the ‘edit colours’ tool, extract the red, green and blue (R,G,B) intensities from the pixel.

6. Repeat the process for a pixel from each of the centre and right of the sample and 3 from the white paper each to the left and right of the sample flask.

7. Calculate the green pixel intensity for each of the selected pixels:

$$Green\ Pixel\ Intensity\ (GPI) = \frac{G}{R + G + B}$$

8. Subtract the average GPI of the white paper from the average GPI of the sample pixels for each sample.

9. Subtract the average GPI of the media from the average GPI of each sample to obtain a final GPI for each sample

10. Plot average final GPI against average chlorophyll concentration to obtain a standard curve. Fit a line of best fit to the linear portion of the curve. Concentrations above the linear portion will need to be diluted prior to analysis.

11. Samples can then be analysed as above and the chlorophyll concentration estimated from green pixel intensity and the created standard curve.

Chlorophyll quantification by standard analytical method (according to the method of Porra *et al*., 1989):

1. Take 1 ml of sample and pellet (13,000 rpm, 10 minutes) in a microcentrifuge.

2. Decant the supernatant and resuspend the pellet in 200 µl methanol, 800 µl acetone.

3. Vortex to ensure the pellet is fully resuspended.

4. Centrifuge (13,000 rpm, 5 minutes) in a microcentrifuge to pellet cell debris.

5. Decant the supernatant into a glass cuvette and measure absorbance at 663.3, 646.6 and 750 nm against an 80% (v/v) acetone/ 20% (v/v) methanol blank.

6. Calculate chlorophyll concentration according to the following equations:

$$Chl\ a\ (\mu g/ml) = 12.25\ E_{663.6} - 2.55\ E_{646.6}$$

$$Chl\ b\ (\mu g/ml) = 20.3\ 1E_{646.6} - 4.91\ E_{663.6}$$

$$Chl\ a + b\ (\mu g/ml) = 17.76\ E_{646.6} + 7.34\ E_{663.6}$$

where $E_{663.6}$ and $E_{646.6}$ represent absorbances at 663.6 nm and 646.6 nm minus absorbance at 750 nm respectively.

Reference

Porra, R. J., Thompson, W. A., & Kriedemann, P. E. (1989). *Determination of accurate extinction coefficients and simultaneous equations for assaying chlorophylls a and extracted with four different solvents: verification of the concentration of chlorophyll standards by atomic absorption spectroscopy*. Biochemica et Biophysica Acta, **975**, 384-394.
